# Combining Phenolization Treatment with the Mannich Reaction for Modification of Kraft Lignin to Produce Highly Efficient Lignin-Based Nitrogen Fertilizer

**DOI:** 10.3390/polym18111281

**Published:** 2026-05-23

**Authors:** Xinkai Mo, Yingchao Wang, Zhongjian Tian, Xingxiang Ji, Fengshan Zhang, Jingpeng Zhou

**Affiliations:** 1 State Key Laboratory of Green Papermaking and Resource Recycling, Qilu University of Technology (Shandong Academy of Sciences), Jinan 250353, China; 18654613976@163.com (X.M.);; 2 Shandong Huatai Paper Co., Ltd., Dongying 257335, China

**Keywords:** kraft lignin, Mannich reaction, nitrogen slow-release fertilizer, phenolization, amination

## Abstract

In this study, the amination-based modification of kraft lignin (KL) was implemented through phenolization treatment combined with the Mannich reaction to synthesize the aminated lignin (APKL) with high nitrogen content. Afterward, the chemical structural changes and reaction mechanism of KL during the modification process were surveyed in depth using diverse analytical techniques. The results revealed that the phenolization treatment markedly raised the active site number in KL from 5.79 to 25.5 mmol/g, which led to a significant increase in the chemical reactivity of KL. Meanwhile, the amine group was successfully grafted onto the best phenolized kraft lignin (PKL) after the Mannich reaction. Furthermore, the effects of amination reagent, reactant mass ratio, temperature and time on the nitrogen content of APKL were systematically examined to optimize the reaction conditions for amination. Using FTIR, molecular weight and elemental analyses, the optimal amination conditions were determined as a reaction temperature of 75 °C, reaction time of 3 h and PKL_6_/arginine/formaldehyde mass ratio of 3:21:28. Under these parameters, APKL_10_ with a higher nitrogen content of 19.2% and lower C/N ratio of 2.46 was acquired. In addition, TG and SEM results revealed that the obtained APKL_10_ possessed a flake-like structure and outstanding thermal stability, which was beneficial for its subsequent application as a slow-release soil fertilizer. More importantly, the soil column leaching test confirmed that the as-prepared APKL_10_ had excellent nitrogen slow-release properties in the soil. As a result, this kraft lignin derivative generated by phenol treatment followed by amination-based modification could serve as an efficient nitrogen fertilizer, providing a long-term nitrogen source for plant growth in soil.

## 1. Introduction

With the thriving development of global industry and continuous expansion of population size, fossil resources had been trapped in the dilemma of excessive exploitation and over-consumption in the past few decades, which brought about a series of environmental problems such as air pollution, global warming, ecosystem destruction and so forth. Thus, it is urgent to exploit green and sustainable new energy sources that can replace fossil resources, which will greatly promote the industrial development of renewable biomass resources [1]. As a natural and renewable resource, lignocellulosic biomass is assumed to be a promising alternative due to its abundant reserves, environmental friendliness and carbon neutrality [2]. In recent years, with the gradual emergence of the concept of efficient utilization of biomass resources, the modern pulp and paper industry is rapidly developing [3]. In the meantime, the large amount of black liquor derived from the pulping process is also generated as a by-product. Most of the black liquor is directly burned to offer heat energy for the pulp mills, while only a small portion is effectively utilized to extract valuable lignin [4]. Regretfully, the extracted lignin has various impurities, poor chemical reactivity and a complex structure, which profoundly limit its subsequent application [5]. For this reason, over the last 10 years, intensive efforts have been devoted to the value-added development of extracted lignin to improve its availability and profitability.

In fact, previous studies on the high-value utilization of lignin had confirmed that the kraft lignin (KL) extracted from black liquor had enormous potential for the fabrication of diverse lignin-based multifunctional composites [6,7,8,9]. For instance, KL could be liquefied by acid catalysis to produce biobased KL-polyurethane foam with a rigid structure and excellent hydrophilicity due to the existence of multitudinous hydroxyls in KL molecules [10]. Furthermore, KL contains abundant methoxy and phenolic hydroxyl functional groups, which are capable of hindering the oxidative propagation reaction via hydrogen donation, thereby revealing KL’s fine antioxidant capacity [11,12]. Hence, KL could be applied as an antioxidative agent to prepare various antioxidant composites including film, hydrogel and so on [13,14,15]. In addition to its antioxidant activity, the rich phenolic hydroxyls and rigid benzene ring structures of KL would absorb ultraviolet radiation from sunlight, which enabled it to serve as a functional filler for synthesizing UV-blocking materials [16,17]. Additionally, KL can be utilized for fabricating multifarious adsorbents to remove dyes or heavy metals in wastewater owing to its many advantages such as low price, eco-friendliness, renewability and biodegradability [18,19,20,21]. More importantly, as a natural urease inhibitor, KL can impede the urease activity in soil, which effectively decelerates the decomposition speed of urea and prolongs its retention time in the soil [22]. Concurrently, KL is also a favorable precursor for humic acid, which can be completely degraded into humus by microorganisms in the soil, thus significantly increasing soil organic matter content and enhancing soil fertility [23]. These superior characteristics highlight KL as an ideal feedstock for manufacturing slow-release nitrogen fertilizer [24,25].

Recently, numerous researchers have exploited a series of slow-release fertilizers utilizing KL as a feedstock through coating, chemical modification and graft polymerization methods involving lignin-based coated fertilizer (LCF) and lignin-based organic nitrogen fertilizer (LONF) [26,27,28]. LCF is produced by coating the surface of fertilizer particles with specially treated KL materials [29]. Unfortunately, the slow-release capability of LCF is constrained by its inferior film-forming performance and poor hydrophobicity. Consequently, in order to achieve a superb slow-release effect, chemical modification technologies such as amination, esterification and polymerization are generally employed to introduce nitrogen into the KL structure and generate LONF [1,30]. Among these techniques, the amination treatment (Mannich reaction) is deemed to be one of the most promising modification technologies, as it can introduce many amine groups into KL molecules [22,31,32]. At present, it has been extensively applied in the modification of KL and synthesis of lignin-based slow-release materials. However, KL has lower chemical reactivity and fewer active sites, which result in less N content (typically ranging from 2.5% to 8.0%) in the aminated KL, thereby profoundly limiting its application in the fertilizer industry. To introduce more amine groups in the Mannich reaction, some studies have conducted activation treatments of KL before amination-based modification, mainly including phenolization [1,31], demethylation [33], and depolymerization [29]. Of these methods, the phenolization treatment is the most effective in increasing the active site content of KL, because it can introduce phenol into the lignin structure, thereby markedly elevating the chemical reactivity of KL [34]. Based on the above literature analysis, this study proposes a scientific hypothesis that targeted phenolization activation can significantly increase the reactive sites of kraft lignin, and the subsequent amination-based modification can achieve efficient nitrogen grafting under mild conditions, which is the fundamental theoretical basis for the rationality of the two-step combined modification strategy. Nevertheless, current studies on the production of lignin-based nitrogen fertilizers via phenolization treatment combined with amination-based modification generally have several limitations [35], such as tedious multi-step operation procedures and harsh reaction conditions (e.g., high temperature and pressure). More crucially, the commonly used amination reagent, ethylenediamine, is a toxic chemical that poses significant safety risks during application, while raising concerns about environmental pollution [36]. To address these issues, this study selected a non-toxic and eco-friendly amination reagent, arginine, to replace ethylenediamine for the synthesis of lignin-based slow-release fertilizer with high nitrogen content using a simple two-step modification strategy.

Herein, KL was firstly activated by phenolization modification under acidic condition; then, the resulting product was grafted with amine groups through the Mannich reaction under alkaline conditions to prepare the aminated phenolized kraft lignin (APKL) with high nitrogen content. Afterward, the chemical structural variations before and after KL modification and the corresponding reaction mechanism were systematically studied by FTIR, XPS, ^31^P, ^1^H, ^13^C and 2D-HSQC NMR analysis. Moreover, the effects of amination reagent, reactant mass ratio, reaction temperature and time on the nitrogen content of APKL were comprehensively examined to optimize the reaction conditions for phenolization and amination. Meanwhile, the physicochemical characteristics of KL, PKL, and APKL were also surveyed using GPC, DTG, SEM and elemental analysis. Furthermore, the soil column leaching test was executed to evaluate the nitrogen release performance of the best APKL in soil. This research offers a significant theoretical foundation and practical methodology for advancing the valorization of kraft lignin.

## 2. Materials and Methods

### 2.1. Materials

The lignin used in this research was extracted from the kraft pulping black liquor of Acacia wood chips through sulfuric acid precipitation followed by purifying and drying as detailed in a previous report [37]. Tetrahydrofuran (THF, 99%), arginine, diethylenetriamine, polyethylene, cyclohexanol (≥98.5%), anhydrous pyridine, deuterated pyridine (D, 99.5%), 2-chloro-4,4,5,5-tetramethyl-1,3,2-dioxaphospholate (TMDP, 97%), 40% dimethylamine aqueous solution, 37% formaldehyde aqueous solution and potassium bromide (KBr, spectrographic grade) were all supplied by Macklin Biochemical Co., Ltd., Shanghai, China. Phenol (≥99%) was bought from Xilong Scientific Co., Ltd., Shanghai, China. Sodium hydroxide (NaOH, ≥97%), acetic anhydride (≥98.5%), sulfuric acid (H_2_SO_4_, 98%) and hydrochloric acid (HCl, 37%) were all purchased from Sinopharm Chemical Reagent Co., Ltd., Shanghai, China. Chromium (III) acetylacetonate, deuterated dimethyl sulfoxide (DMSO-d_6_, 99.8%) and deuterated chloroform (CDCl_3_, 99.8%) were all provided by Sigma-Aldrich company, St. Louis, MO, USA. Dialysis membrane with a molecular weight cut-off of 1000 g/mol was obtained from Spectrum Labs. Inc., San Francisco, CA, USA. All chemicals were used as received without further purification, and deionized water was used in all experiments.

### 2.2. Phenolization Modification of Kraft Lignin

**Figure 1:**
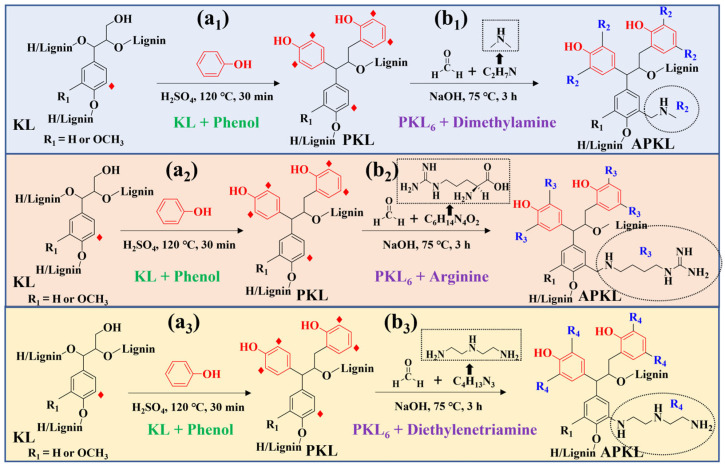
Synthesis mechanism of APKL with high nitrogen content: (**a_1_**–**a_3_**) Phenolization reaction of KL under acidic conditions; (**b_1_**–**b_3_**) Mannich reaction of PKL_6_ under alkaline conditions.

Based on earlier studies [1,29], the phenolization modification of kraft lignin (KL) was implemented in acidic conditions to enhance its chemical reactivity, thereby providing more active sites for subsequent amination treatment. Herein, 7 sets of experiments were designed in which 1 g KL was first dissolved in a certain amount of phenol solution, and the mass ratio of KL and phenol were 1:1, 1:1.5, 1:2, 1:2.5, 1:3, 1:3.5 and 1:4 for PKL_1_, PKL_2_, PKL_3_, PKL_4_, PKL_5_, PKL_6_ and PKL_7_, respectively. Subsequently, 5 mL H_2_SO_4_ was added into the above mixture and then reacted for 30 min at 120 °C. After the reaction, the resulting mixture was slowly poured into HCl aqueous solution with pH = 2 under magnetic stirring to precipitate the phenolized kraft lignin. Later, the resulting precipitant was sequentially filtered, washed and dried to acquire the phenolized kraft lignin (PKL). The reaction mechanism of the phenolization modification for kraft lignin is displayed in Figure 1a_1_–a_3_.

### 2.3. Amination-Based Modification of PKL

As seen in Figure 1b_1_–b_3_, the amination-based modification of PKL was achieved by Mannich reaction in alkaline conditions [1]. In this experiment, the PKL_6_ sample with the best phenolization effect was selected for subsequent Mannich reaction. Simply, 1.5 g of PKL_6_ was dissolved in 20 mL of 0.4 mol/L NaOH solution, followed by stirring for 15 min at 300 rpm. Next, a certain amount of amination reagent (dimethylamine, diethylenetriamine or arginine) and 37% formaldehyde aqueous solution were successively added into the above solution (Tabel S1). Afterward, the resulting mixture was allowed to react at a specific temperature (60, 75 or 90 °C) for a specific time (3, 4 or 5 h) with magnetic stirring at 400 rpm. After completing the reaction, the mixture was cooled to room temperature and then dialyzed using a dialysis bag for 48 h. Finally, the resulting product was freeze-dried to obtain the aminated phenolized kraft lignin (APKL). As a control, 1.5 g KL was dissolved in 20 mL of 0.4 mol/L NaOH solution; then, 14 g of 37% formaldehyde aqueous solution and 10.5 g arginine were mixed into the above solution, followed by reacting at 75 °C for 3 h to synthesize the aminated kraft lignin (AKL). The detailed reaction conditions for the amination-based modification of PKL_6_ or KL are listed in Table S1.

### 2.4. Characterization of KL, PKL and APKL

The chemical structures of KL, PKL and APKL were characterized by FTIR, XPS, ^31^P, ^13^C, ^1^H and 2D-HSQC NMR spectroscopy. The molecular weight and polydispersity index of KL, AKL and APKL were assessed using gel permeation chromatography at room temperature according to our previous reports [37,38]. The contents of C, N, H in KL, AKL and APKL samples were mensurated by an elemental analyzer via the combustion method [39]. The thermal properties of KL, PKL_6_ and APKL_10_ were surveyed by a thermogravimetric analyzer under N_2_ atmosphere. The surface morphologies of KL, PKL_6_ and APKL_10_ samples were observed using a scanning electron microscope with 5 kV accelerating voltage. The specific testing procedure is detailed in the Supporting Information.

### 2.5. Soil Column Leaching Experiment

To examine the nitrogen release properties of APKL_10_ in soil, a soil column leaching experiment was executed based on the method described in the literature [1,40]. Briefly, the soil was first gathered from a farmland surface of 0–20 cm depth and then sieved through a 10-mesh sieve (pore size 2 mm) to remove small stones and plant roots, followed by air-drying for 5 days. Later, PVC pipes with a height of 25 cm and an inner diameter of 9.5 cm were used to construct the soil columns. On the basis of 1.5 g/cm^3^ soil bulk density, 850 g of air-dried soil was uniformly filled in the bottom of the column as the lower layer. Subsequently, APKL_10_ or urea was thoroughly mixed with 650 g of air-dried soil, respectively, and then filled in the upper part of the column as the top layer (Figure S3). The equivalent dosage of 100 mg·N/kg air-dried soil was utilized for different experiments. Furthermore, an appropriate amount of quartz sand was used to coat the surface of each soil column to reduce environmental interference. Meanwhile, we also conducted a blank control experiment without adding any fertilizer under the same conditions. Each experiment was conducted three times, and the results were averaged.

The soil column was fully wetted with deionized water until no free water seeped out, and then left to equilibrate for 24 h. Throughout the experiment, the soil moisture content was kept at about 75% by adding H_2_O. All soil columns were placed at room temperature for incubation. After 1, 4, 7, 13, 19, 28, 48 and 60 days, 200 mL H_2_O was supplemented into each soil column for leaching, and the resulting leachate was collected, followed by filtering and storing at −25 °C for subsequent testing. The NH_4_^+^ and total nitrogen contents of leachate were measured using an elemental analyzer. The nitrogen release performance of APKL_10_ in the soil was evaluated by calculating the cumulative leaching amount of NH_4_^+^-N and total nitrogen.

## 3. Results and Discussion

### 3.1. Chemical Structure Analysis of PKL

To improve the chemical reactivity of kraft lignin and augment its active site content, KL was modified through phenolization treatment under acidic conditions so as to introduce more amine groups in the subsequent Mannich reaction (Figure 1). Herein, we designed seven different experiments to explore the optimum reaction mass ratio of KL and phenol. The chemical structural features of KL and PKL were investigated in detail using FTIR, XPS and NMR spectroscopy, and the results were as follows.

**Figure 2:**
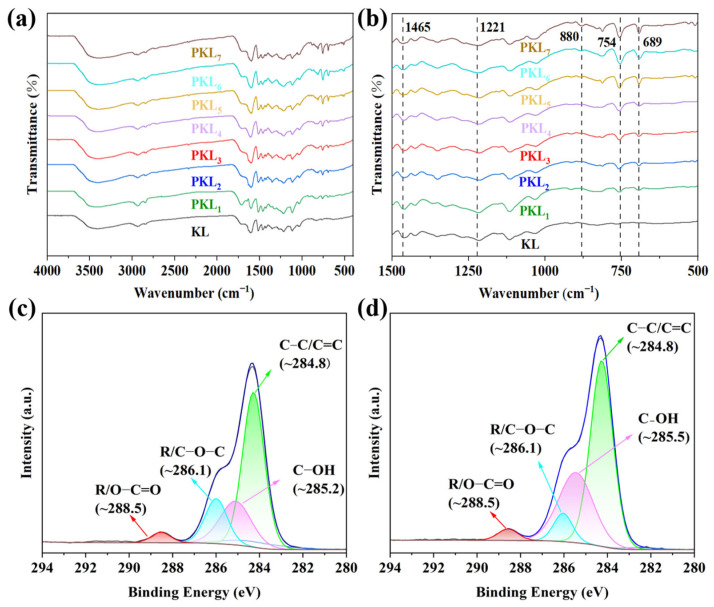
FTIR spectra of KL and all PKL samples at (**a**) 4000–400 cm^−1^ and (**b**) 1500–500 cm^−1^. XPS high-resolution C1s spectra of (**c**) KL and (**d**) PKL_6_.

To preliminarily study the phenolization-based modification effect of kraft lignin, FTIR analysis was first conducted on KL and PKL. Figure 2a,b display the FTIR spectra of KL and PKL samples. As seen, all FTIR spectra showed a broad absorption peak in the range of 3600 and 3180 cm^−1^, which was relevant to the stretching vibrations of aliphatic or aromatic hydroxyl groups [41]. The weak peaks around 2850 cm^−1^ and 2922 cm^−1^ were associated with the symmetric and asymmetric stretching vibrations of C-H in methylene and methyl groups [42]. Moreover, the characteristic peaks at about 1607 and 1519 cm^−1^ were interpreted as the skeleton vibrations of benzene rings [43], while those at 1465 and 880 cm^−1^ were assigned to the C-H bending vibrations and out-of-plane deformation vibrations, respectively [44]. In addition, some absorption bands related to lignin molecules were identified at 1329 and 1273 cm^−1^ (Figure 2b), which were ascribed to the syringyl (S) and guaiacyl (G) structural units in the aromatic skeleton of lignin, respectively [45,46]. The appearance of these characteristic peaks demonstrated that the phenolization-based modification did not alter the basic skeletal structure of KL. Furthermore, compared to FTIR spectra of KL, new peaks around 754 cm^−1^ and 689 cm^−1^ emerged in the FTIR spectra of PKL, derived from the substitution reaction of phenol and kraft lignin side-chain hydroxyl groups [47]. Also, the intensities of these peaks were gradually heightened with the rising mass ratio of KL and phenol. When the mass ratio was 1:3.5, the intensity of the new absorption peaks reached their maximum (Figure 2b). These data indicated that phenol was successfully grafted onto the KL side chains, while the grafting degree of the PKL_6_ sample was the highest.

Later, differences in the chemical structures of KL and the optimized PKL_6_ were deeply investigated through XPS analysis. As depicted in Figure S2, the signals of O1s and C1s were detected in the full XPS spectra of KL and PKL_6_. To further plumb the chemical state of carbon atoms, high-resolution XPS analysis was performed on C1s, and the results are revealed in Figure 2c,d. It could be seen that the C1s spectra of KL and PKL_6_ were deconvoluted into four peaks at 284.8 eV, 285.2 eV, 286.1 eV and 288.5 eV, corresponding to C-C/C=C, C-OH, R/C-O-C and R/O-C=O groups, respectively [48,49]. In contrast with KL, PKL_6_ exhibited an elevated C-OH peak area and a reduced R/C-O-C peak area, proving that the phenolization reaction occurred on the side chains of kraft lignin, which was in accordance with the aforementioned FTIR results. This phenolization treatment led to a significant increase in the content of phenolic hydroxyl groups in PKL_6_, while its methoxyl content was remarkably diminished. Moreover, the phenolization-based modification also introduced some new benzene ring structures into kraft lignin molecules, which also caused an increase in the C-C/C=C peak area in the high-resolution C1s spectra of the PKL_6_ sample (Figure 2d). More importantly, the C-OH peak of PKL_6_ obtained after phenolization shifted towards higher binding energy (285.2 eV → 285.5 eV), suggesting the generation of more phenolic hydroxyl groups with different chemical environments in PKL_6_. These findings were indicative of the successful phenolization reaction between kraft lignin and phenol.

Further, the changes in various hydroxyl contents before and after kraft lignin phenolization were surveyed by ^31^P NMR spectroscopy. It can be seen from Figure S3 that KL contained three types of phenolic hydroxyl groups (syringyl-OH, guaiacyl-OH, *p*-hydroxyphenyl-OH), and the contents of S-OH and G-OH were much higher than that of H-OH. Specifically, the greatest content of S-OH was 9.29 mmol/g, followed by G-OH at 5.46 mmol/g and the lowest content of H-OH at 0.55 mmol/g. As is known to all, there are two active sites in H-OH, one active site in G-OH, and no active site in S-OH [1] This signified that the chemical reactivity of KL was relatively low, which was unfavorable for the subsequent amination-based modification. So, prior to amination, we performed phenolization treatment on the kraft lignin to raise its number of active sites (Figure 1a_1_–a_3_). By comparing the ^31^P NMR spectra of PKL samples fabricated at different mass ratios of KL and phenol, it was found that the signal at 137.5–138.2 ppm in the PKL sample became stronger than that of KL. This finding illustrated that the H-OH content of PKL was sensibly elevated after phenolization treatment. Conversely, the signal intensity of PKL was dramatically weaker than that of KL between 146.1 ppm and 148.9 ppm (Figure S3), principally owing to the replacement of aliphatic hydroxyls by phenol during the phenolization process [31]. Meanwhile, the signal intensity of the non-condensed G-OH (G_NC_-OH) of PKL in the range of 138.9–140.4 ppm was slightly reduced in comparison with KL, which was correlated with the condensation reaction of G_NC_-OH occurring under acidic conditions [50,51]. Furthermore, no residual phenol signal was discovered in the ^31^P NMR spectra of all PKL samples, confirming that the phenolization-based modification had been successfully accomplished.

**Figure 3:**
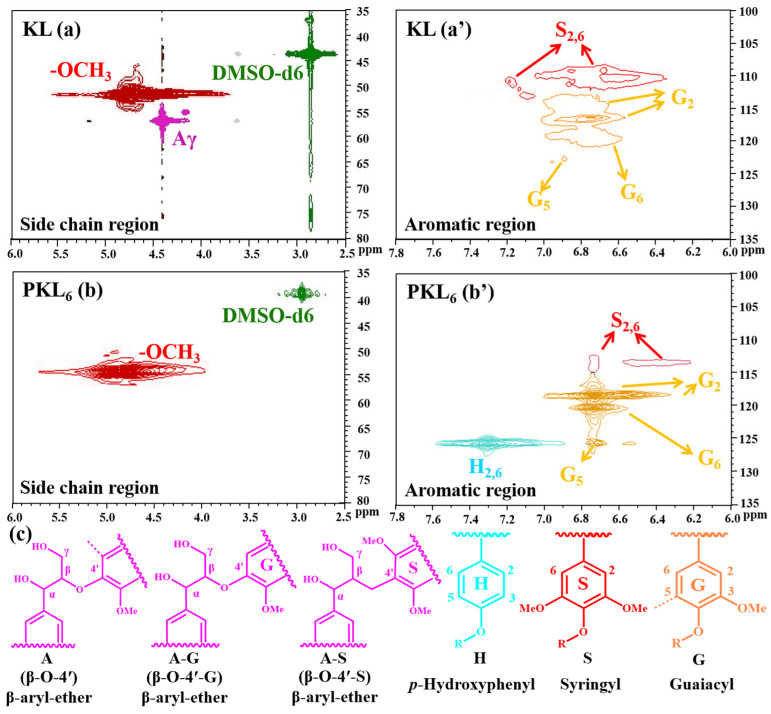
The side chain and aromatic regions in the 2D-HSQC NMR spectra of (**a**,**a’**) KL and (**b**,**b’**) PKL_6_, as well as their main substructures (**c**).

In addition, the above conclusion was further validated by 2D-HSQC NMR analysis. Figure 3 exhibits the 2D-HSQC NMR spectra of the optimal PKL_6_ and KL in the side-chain region (δ_C_/δ_H_ 35–80/2.5–6.0 ppm) and aromatic region (δ_C_/δ_H_ 100–135/6.0–7.8 ppm), along with the chemical substructures of lignin macromolecules. From the side-chain region of KL (Figure 3a,a’), the characteristic signal of methoxy (-OCH_3_) was detected at δ_C_/δ_H_ 51.2/4.78 ppm, while a typical cross-signal referring to β-O-4′ substructure was noticed at δ_C_/δ_H_ 57.8/4.42 ppm (C_γ_-H_γ_, A_γ_) [43,52,53]. However, after phenolization treatment, the β-O-4′ signal disappeared, implying that the phenolic reaction occurred in the side-chain region of KL. Moreover, some cross signals of guaiacyl (G) and syringyl (S) units were visibly observed in the aromatic regions of KL and PKL_6_ (Figure 3). Specifically, the S unit was recognized by the correlations at δ_C_/δ_H_ 113.8/6.53 ppm (C_2,6_-H_2,6_, S_2,6_) [37]. Concurrently, the G unit was characterized by cross-peaks at δ_C_/δ_H_ 121.8/6.78 ppm (C_6_-H_6_, G_6_), δ_C_/δ_H_ 123.8/6.88 ppm (C_5_-H_5_, G_5_) and δ_C_/δ_H_ 117.8/6.75 ppm (C_2_-H_2_, G_2_) [54,55]. More importantly, a new cross signal located at δ_C_/δ_H_ 126.6/7.28 ppm (H_2,6_) was discovered in PKL_6_ (Figure 3b,b’), which proved that phenol had successfully reacted with the KL side chains. In addition, the intensity of the S_2,6_ signal in PKL_6_ was weaker than that in KL, and this signal evidently underwent a low-field shift after phenolization-based modification. This was because the phenolization reaction arose in the KL side chain, which affected the chemical environment of S_2,6_. These results again attested to the successful completion of the phenolization reaction.

**Figure 4:**
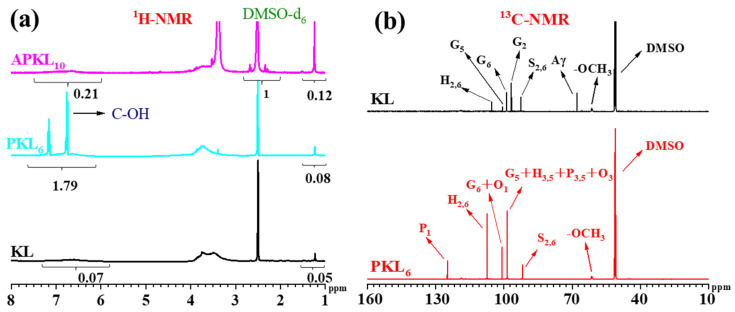
(**a**) ^1^H NMR spectra of KL, PKL_6_ and APKL_10_. (**b**) ^13^C NMR spectra of KL and PKL_6_.

Next, ^1^H NMR analysis was also performed on KL and PKL_6_ to obtain more evidence of the successful introduction of phenol. As shown in Figure 4a, the signal intensity of aromatic protons (6.2–7.7 ppm) in the ^1^H NMR spectra of PKL_6_ displayed a remarkable enhancement after phenolization treatment, suggesting that KL and phenol had undergone the phenolization reaction. It is noteworthy that the signal intensity of α-H and γ-H (3.1–3.98 ppm) sensibly dropped in the ^1^H NMR spectra of the PKL_6_ sample (Figure 4a), which explained that the phenolization reaction occurred on the side chain of KL (Figure 1a_1_–a_3_). To gain a deeper understanding of the chemical structural variations in the kraft lignin during the phenolization reaction, we subsequently performed ^13^C NMR analysis on KL and PKL_6_ samples. As represented in Figure 4b, a basic signal related to -OCH_3_ appeared at 60.8 ppm in the ^13^C NMR spectra of KL and PKL_6_. Meanwhile, several typical signals related to G, H, and S units were clearly distinguished in KL and PKL_6_, such as S_2,6_, G_2_, G_5_, G_6_, and H_2,6_ [56]. However, the intensities of these signals in PKL_6_ were prominently stronger than in KL. This was because the new signals originating from the introduced phenol groups partially overlapped with the original KL signals [1]. In addition to the above signals, an evident signal assigned to β-O-4′ (C_γ_-H_γ_, A_γ_) was also identified at 65.9 ppm in KL (Figure 4b). It was noted that this signal vanished after phenolization, which indicated that the phenolization reaction arose in the KL side chain. Additionally, some new characteristic signals at 98.7 ppm (G_5_ + H_3,5_ + P_3,5_ + O_3_), 101.8 ppm (G_6_ + O_1_), and 128.2 ppm (P_1_) appeared in the ^13^C NMR spectra of PKL_6_, associated with the introduction of new groups by phenolization-based modification [1]. These results were in good concordance with the above-mentioned FTIR, XPS, ^31^P, ^1^H and 2D-HSQC NMR analyses, thereby further verifying the successful grafting of phenol onto kraft lignin side chains.

The above-mentioned FTIR, XPS, ^1^H NMR, ^13^C NMR and 2D-HSQC NMR results fully confirmed the successful introduction of phenol into the KL structure. Afterwards, in order to investigate the optimal reaction conditions for phenolization-based modification, the amounts of diverse hydroxyl groups and active sites in KL and PKL samples were quantitatively calculated on the basis of ^31^P NMR spectra, and the obtained data are listed in Table S2. Based on previous reports [1,50], this research selected 0.5 h and 120 °C as the optimum phenolization time and temperature. Furthermore, the influences of different mass ratios of KL and phenol on the extent of lignin phenolization were systematically examined in this study. It can be seen from Table S2 that after phenolization treatment, the content of aliphatic hydroxyl (Al-OH) in PKL gradually decreased with the rising mass ratio of KL and phenol, whereas the content of *p*-hydroxyphenyl hydroxyl (H-OH) presented an initial increase followed by a decrease. When the KL and phenol ratio was 1:3.5, the H-OH content reached its highest value of 11.01 mmol/g (Table S2), which corresponded to the PKL_6_ sample. Further increasing the dosage of phenol to a mass ratio of 1:4 (PKL_7_) clearly reduced the H-OH content, to 5.99 mmol/g. This variation demonstrated that an appropriate phenol dosage facilitated the introduction of phenol units into lignin molecules, while excessive phenol was not conducive to the phenolization reaction. Accordingly, the PKL_6_ sample exhibited the maximum phenolization degree among all PKL samples and possessed numerous phenol units. Additionally, a slight improvement in the amounts of G_C_-OH and S-OH along with a decline in the amount of G_NC_-OH were observed in all PKL samples in comparison with KL, which implied that the condensation reaction happened in the process of phenolization under acidic conditions [57]. Also, the active site numbers in KL and PKL were calculated according to a method established in an earlier study [29], as tabulated in Table S2. Notably, as the mass ratio of KL and phenol increased, the active site number in PKL first increased and then decreased, which was perfectly consistent with the change in H-OH content. The largest amount of active sites (25.5 mmol/g) was achieved at a mass ratio of 1:3.5, representing a 277.3% increment in the optimal values compared to a previous study [1]. Continued augmentation of the mass ratio of KL and phenol to 1:4 led to a decrease in the number of active sites instead of a rise (Table S2). This phenomenon showed that the phenolization active sites had reached saturation at a mass ratio of 1:3.5. As a result, the PKL_6_ sample with the best phenolization effect was chosen for subsequent amination reaction. 

### 3.2. Chemical Structure Analysis of APKL

**Figure 5:**
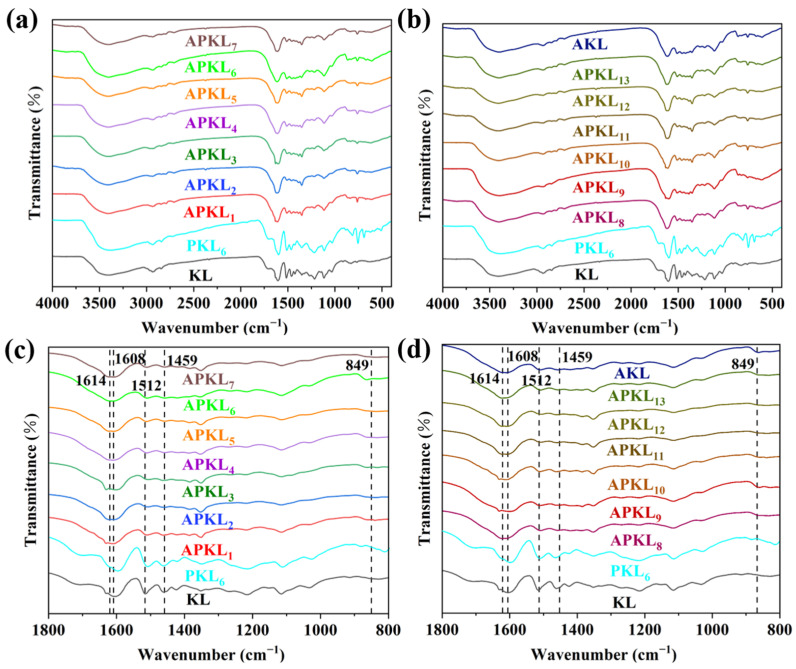
FTIR spectra of KL, PKL_6_ and APKL_1_-APKL_7_ samples at (**a**) 4000–400 cm^−1^ and (**c**) 1800–800 cm^−1^. FTIR spectra of KL, PKL_6_, AKL and APKL_8_-APKL_13_ samples at (**b**) 4000–400 cm^−1^ and (**d**) 1800–800 cm^−1^.

Next, we conducted the amination-based modification on the optimally phenolized kraft lignin (PKL_6_) through Mannich reaction under alkaline conditions to synthesize the aminated PKL_6_ (APKL). In this experiment, 13 types of APKL samples were obtained using various amination reagents, including dimethylamine, diethylenetriamine and arginine, along with different reaction conditions. To tentatively evaluate the level of successful PKL_6_ amination-based modification, the chemical structures of all APKL samples were analyzed by FTIR spectroscopy, and the results are given in Figure 5. It can be observed from Figure 5 that all APKL samples displayed several typical characteristic peaks of kraft lignin [58]. For instance, the wide adsorption band within 3680–3200 cm^−1^ originated from the hydroxyl vibration of aromatic or aliphatic structures [59]. The stretching vibration of the lignin aromatic skeleton appeared at about 1608, 1512 and 1459 cm^−1^ [60]. Further, the peak values around 1327 and 1272 cm^−1^ were correlated with syringyl (S) and guaiacyl (G) groups in the aromatic structure, respectively [57], while that at 1119 cm^−1^ was explained by the ester bond vibration of lignin molecules [61]. This clarified that the amination-based modification did not destroy the skeletal structure of the kraft lignin. Although the aromatic skeleton of KL was not damaged, the intensity of some characteristic peaks varied significantly (Figure 5). As an example, the intensities of adsorption peaks at 2931 cm^−1^ and 2845 cm^−1^, related to C-H stretching vibrations of methyl and methylene groups, were markedly enhanced after amination-based modification [38,62,63], particularly in the FTIR spectra of the APKL_10_ sample. This was because amination reagents containing methylene and methyl were successfully introduced in the Mannich reaction [1]. Furthermore, due to the Mannich reaction occurring on the aromatic ring of PKL_6_, the peak intensity of the aromatic C-H vibration (usually located at 1608, 1512, 1459 and 849 cm^−1^) in APKL samples was prominently lower than that of PKL_6_ and KL. Apart from these peaks, a new absorption peak at 1614 cm^−1^ was detected in the FTIR spectra of all APKL samples and ascribed to the bending vibration of N-H [61]. This demonstrated that the amine group had been successfully grafted onto the PKL_6_ molecule through the Mannich reaction. Simultaneously, the appearance of N1s in the XPS spectra of the APKL_10_ sample (Figure S2) also illustrated the presence of amine in the APKL_10_ structure, thereby further corroborating the successful amination-based modification of PKL_6_. In addition, to quantitatively characterize the surface chemical composition and verify the modification efficiency, the atomic percentages of C, O and N elements in KL, PKL_6_ and APKL_10_ samples were assessed using XPS, and the obtained results are listed in Table S3. As seen, the surface of KL was mainly composed of carbon and oxygen elements, with atomic contents of 72.5% and 27.1%, respectively, while the nitrogen content was only 0.4%. After phenolization treatment, the O content of PKL_6_ rose to 29.3%, and the corresponding O/C atomic ratio was elevated from 0.37 to 0.42, which quantitatively confirmed that abundant phenolic hydroxyl groups were successfully introduced into lignin molecules. Most significantly, the nitrogen atomic percentage of APKL_10_ increased dramatically to 15.3% after the Mannich reaction (Table S3). Such an obvious improvement in surface nitrogen content was highly consistent with the total nitrogen content determined by subsequent elemental analysis, sufficiently proving that nitrogen-containing functional groups were effectively grafted onto the surface of PKL_6_.

**Figure 6:**
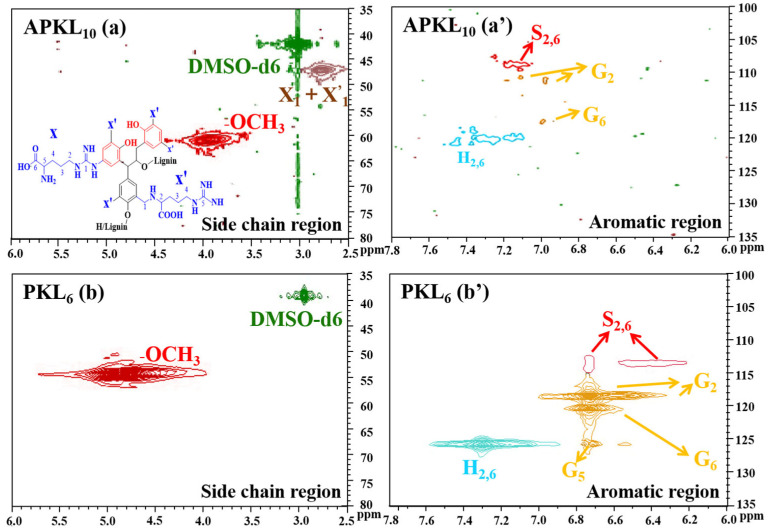
The side-chain and aromatic regions in the 2D-HSQC NMR spectra of (**a**,**a’**) APKL_10_ and (**b**,**b’**) PKL_6_.

Additionally, ^1^H NMR spectra of KL, PKL_6_ and APKL_10_ samples are depicted in Figure 4a to supply more information on the successful grafting of amine groups. It is worth noting that the signal intensity of the aromatic region in the ^1^H NMR spectra of PKL_6_ was dramatically higher than that of KL. Yet, after amination-based modification, a remarkable reduction in this signal was discovered in the ^1^H NMR spectra of APKL_10_, which indicated that the amination reagent had reacted with the aromatic ring of PKL_6_. Furthermore, evident new signals present in the 2.1–2.9 ppm region of the APKL_10_ sample arose from the grafted amine groups. Later, in order to explore the reaction mechanism for the amination-based modification of PKL_6_ in depth, 2D-HSQC NMR analysis was also performed on the PKL_6_ and APKL_10_ samples. Comparing the 2D-HSQC NMR spectra of PKL_6_ and APKL_10_ in the side chain and aromatic regions, several key variations were observed, as shown in Figure 6. For example, a new signal, X_1_ + X’_1_ (δ_C_/δ_H_ 47.2/2.85 ppm), appeared in the side chain region of APKL_10_ (Figure 6a), suggesting that the amine group was successfully introduced into the PKL_6_ structure through the Mannich reaction. As revealed in Figure 6b’, the signal belonging to G_5_ almost completely disappeared in the aromatic region of APKL_10_, while the S_2,6_, G_2_ and G_6_ signals underwent a marginal high-field shift after amination-based modification. In the process, the intensity of the H_2,6_ signal in APKL_10_ was visibly weakened compared with that in PKL_6_. These results showed that the Mannich reaction primarily arose at the G_5_ and H_2,6_ positions of PKL_6_, which was consistent with past research [1,64].

### 3.3. Molecular Weight Analysis of APKL

To further investigate the physicochemical characteristics of KL and APKL, their number-average molecular weight (M_n_), weight-average molecular weight (M_w_) and polydispersity index (PDI = M_w_/M_n_) were mensurated by GPC. The M_n_, M_w_ and M_w_/M_n_ values for KL and APKL samples are tabulated in Table 1. It can be seen from Table 1 that the M_n_, M_w_ and PDI of KL were 1434 g/mol, 1798 g/mol and 1.25, respectively. However, after amination-based modification, the molecular weight and polydispersity index of all APKL samples were lower than those of KL. The main reason was that APKL underwent alkaline catalytic hydrolysis during the Mannich reaction, which made APKL with high molecular weight disintegrate into APKL fragments with low molecular weight, finally leading to a diminution in the average molecular weight of APKL [65]. Among all APKL samples, APKL_10_ exhibited the lowest M_w_ and M_n_ of 1251 g/mol and 1138 g/mol, as well as the narrowest polydispersity of 1.10, implying that the Mannich reaction generated the most uniform APKL_10_ product under a PKL_6_/arginine/formaldehyde mass ratio of 3:21:28, a reaction temperature of 75 °C and a reaction time of 3 h. Combined with the above FTIR and NMR results, it was found that APKL_10_ possessed the most desirable amination-based modification effect, which was compatible with the following elemental analysis results. Such moderate molecular weight and narrow molecular weight distribution were conducive to improving material processability, which also laid a favorable structural foundation for its slow-release performance as a nitrogen fertilizer carrier.

### 3.4. Elemental Analysis of APKL

Apart from the aforementioned chemical structure analysis and molecular weight analysis, we also executed elemental analysis on KL, AKL and APKL samples to determine their C, N, H contents. It can be seen from Table 2 that the nitrogen contents of APKL_7_–APKL_13_ samples were prominently higher than those of the APKL_1_–APKL_6_ samples. This was because the arginine contained more nitrogen atoms, in contrast with diethylenetriamine and dimethylamine, which indicated that arginine is an ideal amination reagent for preparing APKL with high nitrogen content. Moreover, comparing the elemental content of APKL_8_ and APKL_9_ samples, as shown in Table 2, it was found that despite the use of more arginine in the APKL_9_ sample, its nitrogen content was only slightly better than that of the APKL_8_ sample. This might be due to the fact that the reactive sites of PKL_6_ molecules were already saturated under the synthesis conditions of APKL_8_, thereby limiting the further improvement of nitrogen content in APKL_9_. Therefore, considering the production costs associated with APKL, the PKL_6_/arginine/formaldehyde mass ratio of APKL_8_ was selected to optimize the temperature and duration of the Mannich reaction. As shown in Table 2, when the reaction temperature was increased from 60 °C to 75 °C, the nitrogen content of the acquired amination product was significantly increased. Nevertheless, after further elevating the temperature to 90 °C, only a minor increment in the N content of APKL_11_ was observed, as shown in Table 2 (from 19.2% to 19.27%). Hence, 75 °C was chosen as the optimal temperature for the Mannich reaction. In addition, comparing the APKL_10_, APKL_12_ and APKL_13_ samples in Table 2 revealed that prolonging the reaction time brought about a visible decline in the nitrogen content of the aminated product. This phenomenon demonstrated that the longer duration had a negative impact on the N content of APKL. Overall, the APKL_10_ sample obtained by the Mannich reaction at 75 °C for 3 h had the best nitrogen content of 19.20%; thus, the APKL_10_ sample was selected to study its nitrogen release ability in soil in subsequent experiments.

To verify the necessity of phenolization treatment to heighten the nitrogen content, a control AKL sample was set up in this work, using the same Mannich reaction conditions (amination reagent, mass ratio, temperature and time) as for the APKL_10_ sample, except that kraft lignin was used instead of PKL_6_. The elemental analysis showed that the N content of AKL was dramatically lower than that of APKL_10_ (Table 2); this was attributed to the inferior chemical reactivity and fewer active sites of KL, as revealed by above-described ^31^P NMR analysis. These results further testified that the phenolization-based modification was a key pretreatment step for efficient amination by enhancing the chemical reactivity of kraft lignin and raising its active site count. On the basis of the above analysis, the optimal process conditions for fabricating APKL through the Mannich reaction were determined, using arginine as an amination reagent, PKL_6_/arginine/formaldehyde mass ratio of 3:21:28, reaction time of 3 h and reaction temperature of 75 °C.

Previous studies have indicated that lignin derivatives are readily decomposed by soil microorganisms, releasing the organic bound nitrogen when the C/N ratio is below 20 [65,66]. Accordingly, in order to assess the biodegradation potential of the as-prepared APKL in the soil, the corresponding C/N ratio was calculated and is summarized in Table 2. The data in Table 2 show that the C/N ratios of all APKL samples were far lower than 20, between 2.46 and 11.34, suggesting that APKL synthesized by the Mannich reaction has good biodegradability and could effectively release nitrogen into the soil to provide sufficient nutrients for plant growth.

In addition, to clarify the application potential of the modified lignin (APKL_10_) as a slow-release fertilizer, its nitrogen content was compared with traditional chemical fertilizers and previously reported lignin-based fertilizers. The obtained results are summarized in Table S4. In general, the aminated lignin materials produced via conventional routes exhibited low nitrogen content ranging from 3% to 12%, which severely limits their practical application as primary nitrogen sources. By contrast, the APKL_10_ sample prepared in this study achieved an outstanding nitrogen content of 19.2%, a 1.5–6.3 fold increase over existing lignin-based fertilizers. Moreover, although commercial urea has a higher theoretical nitrogen content (46%), it is prone to rapid nutrient leaching, thereby presenting low nutrient utilization efficiency. However, the modified lignin product (APKL_10_) in this work had a nitrogen content close to that of commercial ammonium sulfate (21%), which effectively bridged the performance gap between low-nutrient organic carriers and high-pollution inorganic fertilizers. Thus, it is expected to serve as a promising eco-friendly fertilizer for sustainable agricultural development.

### 3.5. Thermal Analysis of KL, PKL and APKL

**Figure 7:**
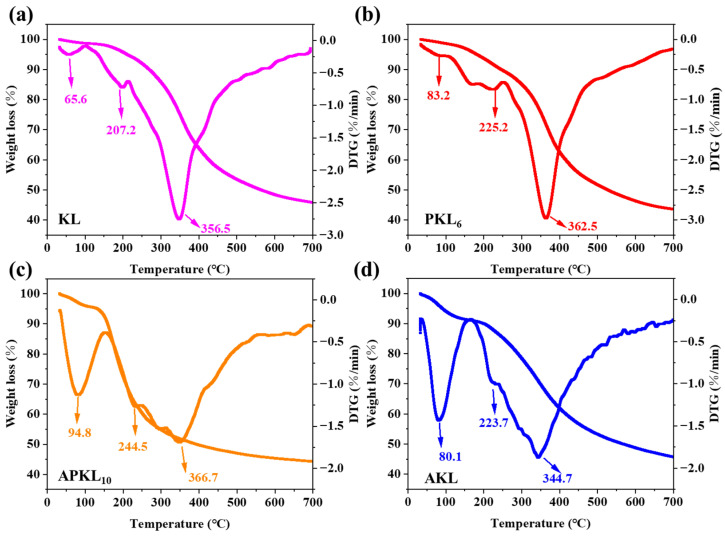
TG and DTG curves of (**a**) KL, (**b**) PKL_6_, (**c**) APKL_10_ and (**d**) AKL samples.

It is well known that the thermal degradation behavior of lignin polymer plays an important role that affects its practical application. Herein, thermogravimetric analysis was implemented to evaluate the thermal properties of KL, PKL_6_, APKL_10_ and AKL samples. The resulting TG and DTG curves are represented in Figure 7, and the thermal degradation parameters are tabulated in Table S5. It can be clearly seen in Figure 7a–c that the thermal weight loss trends of PKL_6_ and APKL_10_ were parallel to that of KL, illustrating that the aromatic structure of KL was not disrupted by phenolization and amination-based modifications, which is in agreement with the results reported in previous literature [1,22]. Notably, the thermal degradation process of these samples could be divided into four principal stages. The first stage was below 140 °C, where the minor weight loss occurred in KL, PKL_6_, APKL_10_ and AKL, due to the evaporation of residual water. The second stage was noticed from 140 °C to 260 °C, which brought about a weight loss of 15–20%. This was mainly attributed to the cleavage of aryl ether bonds as well as decarboxylation reaction, thus resulting in the decomposition and evaporation of lignin fragments with low molecular weight [67,68,69]. The third stage was the severe weight loss at 260–480 °C, which was derived from the breakage of inter-unit linkages in lignin and evaporation of phenol. In this stage, KL, PKL_6_, APKL_10_ and AKL were all decomposed into small molecules and gaseous products [70]. The last stage was above 480 °C, in which the residues were further degraded into gas and char residue [38]. Additionally, as listed in Table S5, the char residue of APKL_10_ was slightly elevated compared to that of KL and PKL_6_, originating from the incorporation of amine groups during the amination process. It was reported that introducing multitudinous amine groups into lignin molecules via the Mannich reaction could enhance the lignin’s thermal properties [71]. Moreover, DTG curves showed that the onset thermal degradation temperature (T_onset_) values for KL and PKL_6_ were 207.2 and 225.2 °C, respectively, while the T_onset_ of APKL_10_ was dramatically increased to 244.5 °C, which was related to the amination-based modification of PKL_6_. At the same time, the maximum weight loss temperature (T_max_) of APKL_10_ (366.7 °C) was also better than those of KL and PKL_6_ (Table S5). In addition, it can be seen from Figure 7d that the T_onset_ of the control AKL sample was only 223.7 °C, and its T_max_ (344.7 °C) was markedly lower than those of PKL_6_ and APKL_10_. This inferior thermal stability of AKL compared to APKL_10_ highlights the critical necessity of phenolization pretreatment. Overall, the aforementioned data indicated that the as-prepared APKL_10_ sample possessed distinguished thermal stability, which was favorable for its subsequent storage as agricultural fertilizer.

### 3.6. Morphology Analysis of KL, PKL and APKL

**Figure 8:**
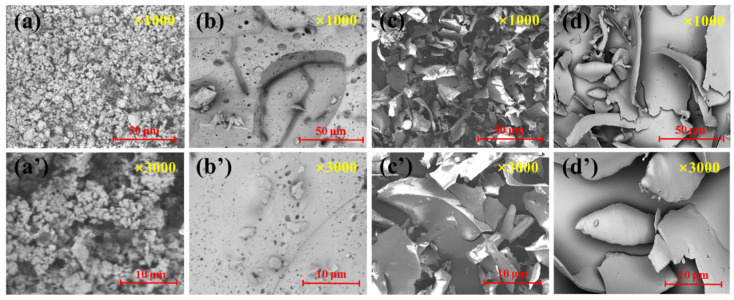
SEM images of (**a**,**a’**) KL, (**b**,**b’**) PKL_6_, (**c**,**c’**) APKL_10_ and (**d**,**d’**) AKL samples at different magnifications.

It is well known that the microstructure of lignin-derived fertilizer has a significant impact on its nutrient release efficiency [72]. For this reason, the surface morphologies of KL, PKL_6_, APKL_10_ and AKL samples were characterized by SEM, and the obtained SEM images are exhibited in Figure 8. As seen in Figure 8a, it was observed that KL presented a loose powdery structure. After phenolization treatment, the resulting PKL_6_ displayed a typical blocky structure, with many pores on its surface (Figure 8b,b’) that might be associated with the introduction of numerous phenolic hydroxyl groups during the phenolization process [73]. These phenolic hydroxyls facilitated the generation of intramolecular hydrogen bonding in KL, making KL macromolecules agglomerate together and form a block-shaped PKL_6_. However, further amination-based modification gave rise to noticeable changes in the microscopic structure of the APKL_10_ sample. After the Mannich reaction of PKL_6_ under alkaline conditions, the surface morphology of PKL_6_ was damaged, forming irregular flakes of various sizes (Figure 8c,c’), which was probably attributable to the -NH_2_ introduced in the Mannich reaction. Furthermore, it can be seen from Figure 8d,d’ that AKL had a surface morphology similar to that of the APKL_10_ sample, whereas its fragmentation degree was obviously lower than that of APKL_10_. This N-containing lignin derivative with remarkably fragmented morphology (APKL_10_) would slowly release nutrients into the soil, while its unique flake-like structure contributed to strengthening the soil permeability and achieving good soil fertilization effects [74].

### 3.7. Nitrogen Release Behavior Analysis of APKL in Soil

**Figure 9:**
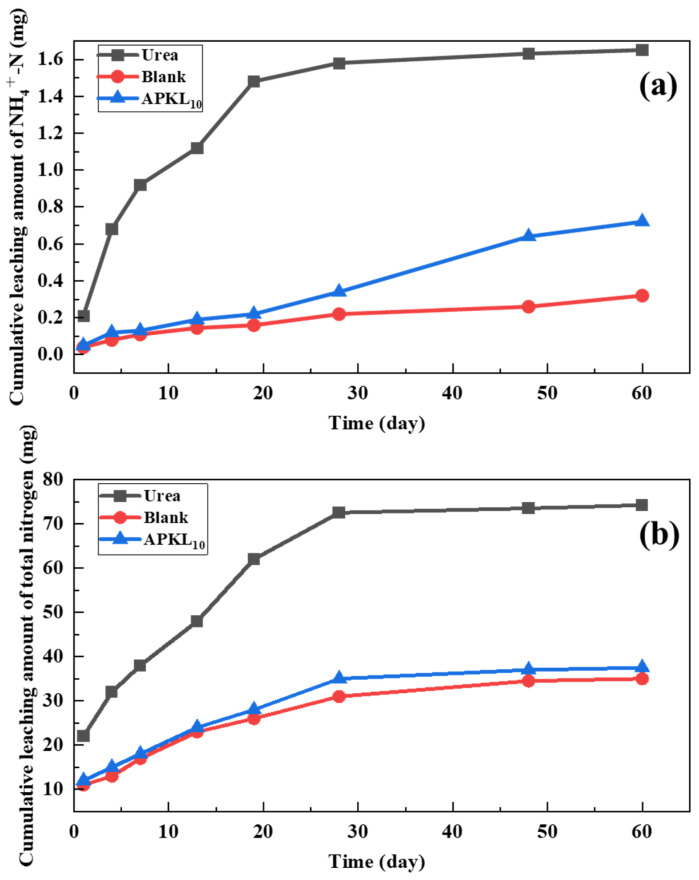
The cumulative amounts of (**a**) NH_4_^+^-N and (**b**) total nitrogen leached by APKL_10_, urea and blank control groups in soil.

Owing to the higher nitrogen content in APKL_10_ synthesized in this study and the C/N ratio much below 20, it can be applied as nitrogen fertilizer in soil. Next, the nitrogen release capacity of the best aminated phenolized kraft lignin (APKL_10_) in soil was examined through a soil column leaching experiment. As presented in Figure 9, the cumulative leached amount of NH_4_^+^ was much lower than that of total nitrogen in all experiments due to serious loss of NH_4_^+^ through volatilization or rapid conversion from NH_4_^+^ to NO_3_^−^ via nitrification in the soil. Furthermore, when urea was employed as a nitrogen fertilizer, most nitrogen was rapidly released into the soil within 28 days. However, this fast release was not only unsynchronized with the plant growth cycle, but also led to a great loss of nitrogen, ultimately causing insufficient nitrogen supply at the later stages of plant growth. Notably, the difference in cumulative NH_4_^+^ release between APKL_10_ and blank control group was not significant in the first 28 days (Figure 9a). This indicated that APKL_10_ had not yet shown a remarkable slow-release effect at this stage, thereby avoiding excessive release and severe loss of nitrogen. Nevertheless, the cumulative amount of NH_4_^+^ leached from the APKL_10_-treated group was obviously augmented after 28 days, whereas only slight changes were noticed in the urea and blank groups in the same period. This finding illustrates that APKL_10_ began to degrade and slowly release elemental nitrogen into the soil after 28 days, which allowed it to serve as a long-term nitrogen source for plant growth. This was in stark contrast to the release pattern of urea. The high nitrogen content and uniform functional group distribution of the APKL_10_ sample were the essential structural factors regulating its staged nitrogen release behavior, which directly determined its excellent slow-release performance. Consequently, it was considered that the as-prepared APKL_10_ had enormous potential for application as a slow-release fertilizer in the agricultural field, which would promote the high-value utilization of kraft lignin.

## 4. Conclusions

This study synthesized a high-nitrogen-containing lignin derivative using kraft lignin (KL) as a raw material through phenolization treatment combined with amination-based modification. Then, the chemical structural variations before and after KL modification and the corresponding reaction mechanisms were systematically investigated by FTIR, XPS, ^31^P, ^1^H, ^13^C and 2D-HSQC NMR analysis. The results showed that the active site number of KL was prominently increased by phenolization treatment, while its chemical reactivity was also obviously enhanced. In the subsequent amination-based modification, the amination reagent type was the key factor affecting the N content in the aminated product, with arginine considered to be the best choice among three amination reagents. Moreover, the optimum aminated phenolized kraft lignin (APKL_10_) was obtained with a PKL_6_/arginine/formaldehyde mass ratio of 3:21:28, reaction temperature of 75 °C and reaction time of 3 h during the Mannich reaction. Also, the obtained APKL_10_ possessed a relatively high nitrogen content of 19.2% and a relatively low C/N ratio of 2.46, which would help it release nitrogen in soil to provide nutrients for plant growth. Additionally, the irregular flake-like APKL_10_ contributed to elevating the soil permeability, enabling it to achieve superior nitrogen slow-release performance in the soil. The findings of this research could promote the application of kraft lignin in the fertilizer industry and prove beneficial for expanding the value-added utilization of kraft lignin.

## Figures and Tables

**Table 1:** The M_w_, M_n_ and M_w_/M_n_ of KL, AKL, PKL_6_ and APKL samples.

Sample Label	M_w_ (g/mol)	M_n_ (g/mol)	PDI = M_w_/M_n_
KL	1798	1434	1.25
PKL_6_	1532	1298	1.18
APKL_1_	1773	1430	1.24
APKL_2_	1609	1330	1.21
APKL_3_	1595	1352	1.18
APKL_4_	1724	1402	1.23
APKL_5_	1518	1320	1.15
APKL_6_	1491	1308	1.14
APKL_7_	1543	1286	1.20
APKL_8_	1630	1325	1.23
APKL_9_	1661	1396	1.19
APKL_10_	1251	1138	1.10
APKL_11_	1277	1140	1.12
APKL_12_	1481	1245	1.19
APKL_13_	1565	1326	1.18
AKL	1559	1247	1.25

**Table 2:** The elemental content of KL, AKL and APKL samples.

Sample Label	Elemental Content (%)			C/N Ratio
	C	N	H	
KL	62.12 ± 0.52	0.33 ± 0.15	5.28 ± 0.21	188.24
APKL_1_	49.21 ± 0.52	4.34 ± 0.15	5.61 ± 0.21	11.34
APKL_2_	48.30 ± 0.52	4.66 ± 0.15	5.60 ± 0.21	10.36
APKL_3_	49.90 ± 0.52	5.31 ± 0.15	5.73 ± 0.21	9.40
APKL_4_	46.26 ± 0.52	6.12 ± 0.15	5.36 ± 0.21	7.56
APKL_5_	46.12 ± 0.52	6.24 ± 0.15	5.21 ± 0.21	7.39
APKL_6_	49.82 ± 0.52	7.01 ± 0.15	5.48 ± 0.21	7.11
APKL_7_	51.91 ± 0.52	11.36 ± 0.15	5.89 ± 0.21	4.57
APKL_8_	44.62 ± 0.52	16.06 ± 0.15	6.37 ± 0.21	2.78
APKL_9_	44.30 ± 0.52	16.15 ± 0.15	6.31 ± 0.21	2.74
APKL_10_	47.17 ± 0.52	19.20 ± 0.15	5.99 ± 0.21	2.46
APKL_11_	47.41 ± 0.52	19.27 ± 0.15	6.49 ± 0.21	2.46
APKL_12_	49.45 ± 0.52	17.32 ± 0.15	6.32 ± 0.21	2.86
APKL_13_	48.28 ± 0.52	17.12 ± 0.15	6.37 ± 0.21	2.82
AKL	49.06 ± 0.52	14.75 ± 0.15	5.99 ± 0.21	3.33

Note: The experimental data are presented as means plus standard deviation (SD).

## Data Availability

The data presented in this study are available on request from the corresponding authors.

## Supplementary Materials

The following supporting information can be downloaded at: https://www.mdpi.com/article/10.3390/polym18111281/s1. Table S1. The specific reaction conditions for the amination modification of PKL6 or KL. Table S2. The contents of various hydroxyl groups in KL and PKL calculated by 31P NMR spectra. Table S3. Atomic percentage and O/C molar ratio of KL, PKL6 and APKL10 samples. Table S4. Performance comparison of the modified lignin-based nitrogen slow-release fertilizer prepared in this work with commercial fertilizers and literature-reported lignin-based fertilizers. Table S5. Thermal degradation parameters of KL, PKL6, APKL10 and AKL samples. Figure S1. Schematic diagram of the equipment used in the soil leaching experiment. Figure S2. Full XPS spectra of KL, PKL6 and APKL10 samples. Figure S3. 31P NMR spectra of KL and all PKL samples.
