# Supplementary material for: Combining Phenolization Treatment with the Mannich Reaction for Modification of Kraft Lignin to Produce Highly Efficient Lignin-Based Nitrogen Fertilizer
Source: Polymers (Basel). 2026 May 23;18(11):1281. doi: 10.3390/polym18111281 (PMC13259078; doi:10.3390/polym18111281)
Supplement: Supplementary file 1 [file polymers-18-01281-s001.zip › polymers-4299251-supplementary.pdf]

Supporting Information:

Combining phenolization treatment with Mannich reaction for the modification of kraft lignin to produce highly efficient lignin-based nitrogen fertilizer

Xinkai Mo[a], Yingchao Wang[a], Zhongjian Tian[a,b,*], Xingxiang Ji[a,*], Fengshan Zhang[b], Jingpeng Zhou[b]

[a] State Key Laboratory of Green Papermaking and Resource Recycling, Qilu University of Technology (Shandong Academy of Sciences), Jinan, 250353, China

[b] Shandong Huatai Paper Co., Ltd., Dongying, 257335, China

[*] Corresponding author:

E-mail addresses: tianzhj@qlu.edu.cn (Z. Tian); xxjt78@163.com (X. Ji).

Experimental Section:

FTIR analysis

Fourier transform infrared (FTIR) spectra of KL, PKL, AKL and APKL were investigated by a FTIR spectrometer (Bruker ALPHA II, Germany) in the wavenumber range of 4000-400 cm^{-1} with a resolution of 4 cm^{-1} for 32 scans. Before testing, 2 mg of the oven-dried lignin powder was milled with 200 mg of the oven-dried KBr powder and pressed into a flake specimen for FTIR analysis.

XPS analysis

XPS test was performed on an X-ray photoelectron spectrometer (XPS, ThermoFisher ESCALABXi+, US) equipped with a monochromatic Al Kα radiation (1486.6 eV) at a base pressure of 5×10^{-10} mbar. Different lignin samples (KL, PKL, APKL) were placed on the platform for XPS analysis. High-resolution spectra of C1s were measured at a pass energy of 50 eV. The binding energy scale was calibrated to the C1 line peak at 284.8 eV. Data analysis and peak fitting were conducted using Avantage software.

NMR analysis

The ^{31}P, ^{13}C, ^{1}H and 2D-HSQC NMR spectra of KL, PKL and APKL were surveyed by a 400 MHz NMR spectrometer (Bruker AVANCE III, USA). To determine the hydroxyl content in KL and PKL samples, the quantitative ^{31}P NMR analysis was conducted according to the modified method reported in the literature [1] Specifically, 20 mg of the oven-dried KL or PKL sample was firstly dissolved in 0.5 mL mixed solution of anhydrous pyridine and deuterated chloroform (1.6:1, v/v) at room temperature. After complete dissolution, 0.1 mL of 4 mg/mL cyclohexanol as an internal standard (IS) and 0.1 mL of 3.6 mg/mL chromium (III) acetylacetonate solution as a relaxation reagent were added in sequence and then sonicated for 5 min to obtain uniform mixture. Subsequently, the mixture was reacted with 0.1 mL of 2-chloro-4,4,5,5-tetramethyl-1,3,2-dioxaphospholate (TMDP) as the phosphorylating reagent for 15 min. Afterward, the resulted mixture was vibrated for 10 min to ensure the completion of phosphorylation reaction. Finally, the as-prepared sample was transferred into a 5 mm nuclear magnetic tube for analysis.

For the 2D-HSQC NMR test, 30 mg of the oven-dried KL, PKL_6 or $APKL_{10}$ was completely dissolved in 0.5 mL of DMSO-d_6 (internal standard TMS) and then stirred overnight at 25 °C followed by ultrasonic and centrifugation. Afterward, the resulting supernatant was transferred into NMR tube for ^{1}H NMR and 2D-HSQC NMR analysis. Besides, the ^{13}C NMR analysis of KL and PKL_6 was also conducted after dissolving 140 mg samples in 0.5 mL DMSO-d_6 with TMS as an internal chemical shift reference. The adjustment parameters were carried out using a standard Bruker pulse sequence in accordance with our previous work[2]. The spectral widths were 8,000 and 30,000 Hz for the ^{1}H and ^{13}C dimensions, respectively. The number of collected complex points was 1024 for the ^{1}H dimension with a recycle delay of 1.5 s. Meanwhile, the number of transients was 256 increments of 64 s, which was recorded in

the ^{13}C dimension. A 90 ° pulse, 0.11 s acquisition time, 1.5 s pulse delay, and 48 scans were followed to acquire 2D-HSQC spectra. The $^{1}J_{C-H}$ of 145 Hz was used in this experiment. Data processing was executed using Bruker Topspin 4.1.1 software and Mestre Nova 14 software.

Thermogravimetric analysis

The thermal stabilities of KL, PKL_6 and $APKL_{10}$ samples were investigated by a thermal gravimetric analyzer (TGA, Q50, USA) according to previous report[3]. Specifically, 5-10 mg of KL or PKL_6 or $APKL_{10}$ sample was heated from 30 °C to 700 °C at a heating rate of 10 °C/min under N_2 atmosphere.

GPC analysis

The weight-average molecular weight (M_w), number-average molecular weight (M_n) and polydispersity index ($PDI=M_w/M_n$) of KL, AKL and APKL were mensurated by a gel permeation chromatography (GPC, Waters e2695, Japan), which was equipped with refractive index detector. Before testing, 50 mg of dried KL, AKL or APKL was dissolved in 4 mL mixed solution of pyridine/acetic anhydride (1:1, v/v) and then stirred at room temperature for 48 h without light radiation. After evaporating excess pyridine and acetic anhydride, 50 mL of HCl aqueous solution (pH=2, 4 ℃) was slowly dropped into the resulting concentrated solution to precipitate the acetylated lignin followed by freeze-drying. Subsequently, the freeze-dried acetylated lignin was dissolved in tetrahydrofuran (THF, 1 mg/mL) and then filtered by a nylon filter with 0.2 μm pore diameter. The solution-state samples were analyzed by a chromatography column (Styragel® HR 4 THF) with each injection volume of 10 μL. The column temperature was kept at 40±0.1 °C and the external UV detector was set at 254 nm. THF was selected as a mobile phase at a flow rate of 0.6 mL/min. Polyethylene was used as a standard sample for calibration [4,5]. All samples were tested in triplicate and the average values were recorded.

Elemental analysis

The elemental analysis was executed for KL, AKL and APKL samples through the combustion method [6]. All samples were firstly oven dried at 105 ℃ prior to analysis. Then, about 2 mg of dried sample was combusted at 1200 ℃ for determining their C, H and N contents by an elemental analyzer (Elementar UNICUBE, Germany). All tests were repeated 3 times, and the average result was reported.

Morphology analysis

The surface morphologies of KL, PKL_6 and $APKL_{10}$ were observed by a scanning electron microscope (SEM, Hitachi TM4000Plus, Japan) with an accelerating voltage of 5 kV. Prior to observation, all samples were sputtered with a thin layer of gold to increase the conductivity for better observation.

Table S1. The specific reaction conditions for the amination modification of PKL_6 or KL.

Amination reagent (g)

Sample label	PKL_6 (g)	Formaldehyde (g)	Dimethylamine (g)	Diethylenetriamine (g)	Arginine (g)	Temperature (℃)	Time (h)
$APKL_1$	1.5	3	2.6	---	---	60	3
$APKL_2$	1.5	6	5.2	---	---	60	3
$APKL_3$	1.5	9	7.8	---	---	60	3
$APKL_4$	1.5	12	---	3.9	---	60	3
$APKL_5$	1.5	14	---	7.8	---	60	3
$APKL_6$	1.5	16	---	11.7	---	60	3
$APKL_7$	1.5	3	---	---	9	60	3
$APKL_8$	1.5	6	---	---	10.5	60	3
$APKL_9$	1.5	9	---	---	12	60	3
$APKL_{10}$	1.5	14	---	---	10.5	75	3
$APKL_{11}$	1.5	14	---	---	10.5	90	3
$APKL_{12}$	1.5	14	---	---	10.5	75	4
$APKL_{13}$	1.5	14	---	---	10.5	75	5
AKL	1.5 KL	14	---	---	10.5	75	3

Note: PKL_6: the phenolizated kraft lignin with the mass ratio of reactants (KL/phenol = 1:3.5). APKL: the aminated PKL_6. AKL: the aminated kraft lignin without phenolization treatment. "---": not available in this set of experiment. All reaction optimization experiments (including temperature, reaction time, and reactant ratios) and subsequent characterizations were performed in independent triplicates (n=3).

Table S2. The contents of various hydroxyl groups in KL and PKL calculated by ^{31}P NMR spectra.

Sample label	^{31}P NMR (mmol/g)					Active sites (mmol/g)
	Al-OH	H-OH	S-OH	G_C-OH	G_{NC}-OH	
KL	5.53±0.12	0.55±0.06	9.29±0.18	0.77±0.05	4.69±0.11	5.79±0.15
PKL_1	3.84±0.09	2.57±0.14	9.31±0.15	0.78±0.06	3.32±0.10	8.46±0.22
PKL_2	3.38±0.11	5.68±0.19	9.30±0.17	0.88±0.05	3.60±0.12	14.96±0.31
PKL_3	3.18±0.08	5.74±0.16	9.43±0.21	0.90±0.07	4.07±0.13	15.55±0.28
PKL_4	3.12±0.13	5.80±0.18	9.32±0.16	0.78±0.05	4.33±0.11	15.93±0.34
PKL_5	3.10±0.10	8.02±0.22	9.41±0.19	0.79±0.06	3.63±0.14	19.67±0.38
PKL_6	2.27±0.09	11.01±0.24	9.84±0.16	0.84±0.07	3.50±0.12	25.50±0.42
PKL_7	0.55±0.05	5.99±0.15	9.87±0.21	0.90±0.06	3.78±0.10	15.76±0.28

Note: KL: kraft lignin. PKL: the phenolizated kraft lignin. Al-OH: aliphatic hydroxyl group. S-OH: syringyl hydroxyl group. H-OH: *p*-hydroxyphenyl hydroxyl group. G_C-OH: the condensed guaiacyl

hydroxyl group. G_{NC}-OH: the non-condensed guaiacyl hydroxyl group. Active sites mean the amounts of available sites on the structure of KL or PKL for subsequent Mannich reaction. All reaction optimization experiments (including temperature, reaction time, and reactant ratios) and subsequent characterizations were performed in independent triplicates (n=3). The experimental data were presented as the mean standard deviation (SD).

Table S3. Atomic percentage and O/C molar ratio of KL, PKL_6 and $APKL_{10}$ samples.

Sample label	C (%)	O (%)	N (%)	O/C ratio
KL	72.5±0.8	27.1±0.6	0.4±0.1	0.37±0.01
PKL_6	70.2±0.9	29.3±0.7	0.5±0.1	0.42±0.02
$APKL_{10}$	65.1±1.0	19.6±0.8	15.3±0.5	0.30±0.02

Table S4. Performance comparison of the modified lignin-based nitrogen slow-release fertilizer prepared in this work with commercial fertilizers and literature-reported lignin-based fertilizers.

Fertilizer type	Nitrogen content	Nutrient release type	Reference
Aminated kraft lignin	5.36-10.18	Slow release	Jiao et al., 2019[7]
Lignin-polyurea hydrogel	8.0-12.5	Controlled release	Gao et al., 2019[8]
Traditional aminated lignins	3.0-8.5	Slow release	Li et al., 2020
Commercial urea	40.0-50.0	Quick release	Standard
Commercial ammonium sulfate	20.0-30.0	Quick release	Standard
**$APKL_{10}$**	**4.34-19.27**	**Slow release**	**This work**

Table S5. Thermal degradation parameters of KL, PKL_6, $APKL_{10}$ and AKL samples.

Sample label	T_{onset} (°C)	T_{max} (°C)	Char residue (%)
KL	207.2±1.7	356.5±2.3	42.8±0.9
PKL_6	225.2±2.6	362.5±2.7	43.5±0.7
$APKL_{10}$	244.5±3.1	366.7±3.9	44.3±1.5
AKL	223.7±2.2	344.7±1.8	43.7±1.2

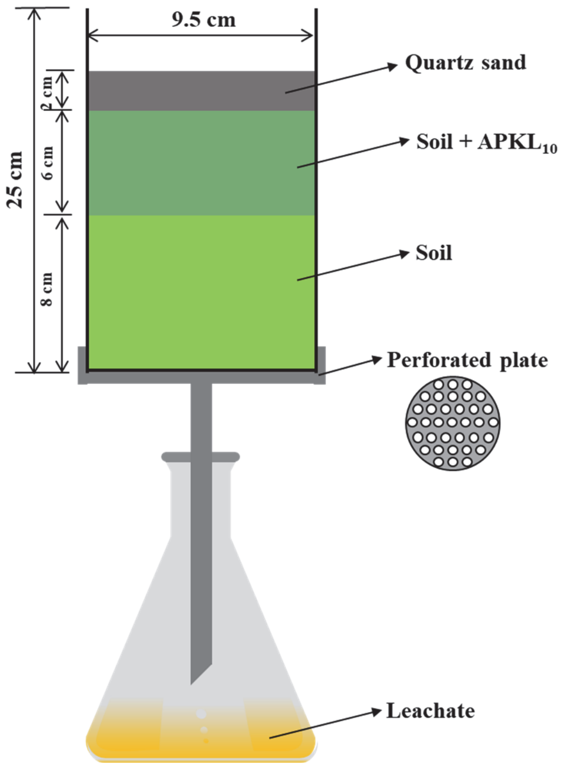

Fig. S1. Schematic diagram of the equipment used in the soil leaching experiment.

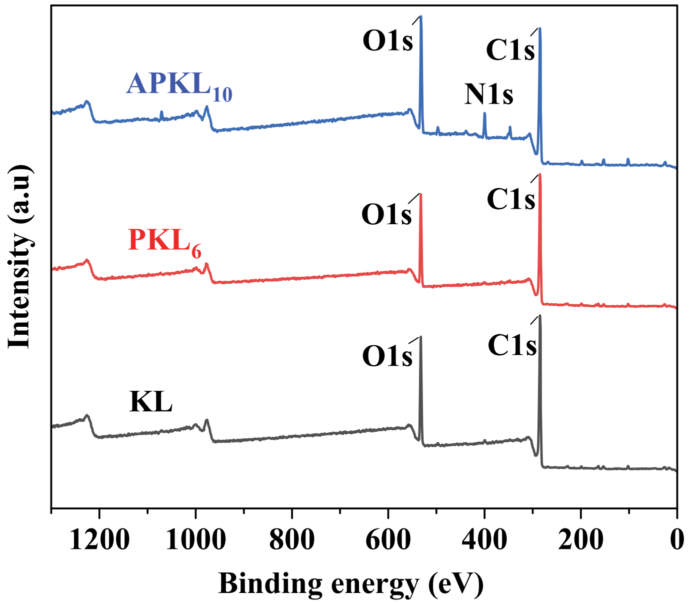

Fig. S2. Full XPS spectra of KL, PKL_6 and $APKL_{10}$ samples.

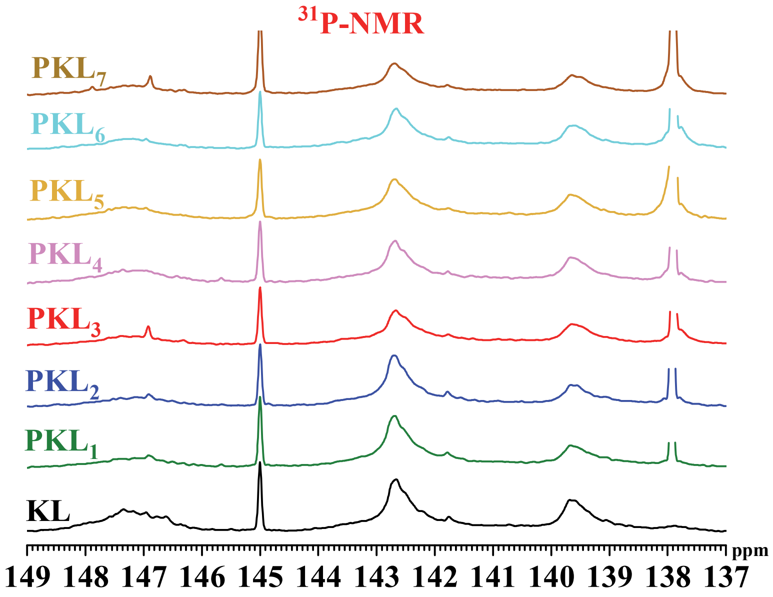

Fig. S3. ^{31}P NMR spectra of KL and all PKL samples.

References

[1]Zhao, L., Wang, Y., Wang, Q., Liu, S., Ji, X., 2024. Lignin nanoparticles produced from wheat straw black liquor using γ-valerolactone. Polymers 16, 49.

[2]Xie, Y., Ji, X., Tian, Z., Wang, Y., Mo, X., Zhang, F., Zhou, J., 2025. Extraction of high-purity lignin from the kraft pulping black liquor by enzyme purification process with alkaline-resistant xylanase and cellulase. Int. J. Biol. Macromol. 295, 139574.

[3]Ghavidel, N., Konduri, M.K., Fatehi, P., 2021. Chemical reactivity and sulfo-functionalization response of enzymatically produced lignin. Ind. Crops Prod. 172, 113950.

[4]Jiang, X., Tian, Z., Ji, X., Ma, H., Yang, G., He, M., Dai, L., Xu, T., Si, C., 2022. Alkylation modification for lignin color reduction and molecular weight adjustment. Int. J. Biol. Macromol. 201, 400-410.

[5]Zhang, J., Tian, Z., Ji, X., Zhang, F., 2023. Light-colored lignin extraction by ultrafiltration membrane fractionation for lignin nanoparticles preparation as UV-blocking sunscreen. Int. J. Biol. Macromol. 231, 123244.

[6]Zhao, L., Diaz-Baca, J., Salaghi, A., Gao, J., Wang, Y., Wang, Q., Fatehi, P., 2023. Cationic tall oil lignin-starch copolymer as a flocculant for clay suspensions. Ind. Crops Prod. 202, 117069.

[7]Jiao, G., Peng, P., Sun, S., Geng, Z., She, D., 2019. Amination of biorefinery technical lignin by Mannich reaction for preparing highly efficient nitrogen fertilizer. Int. J. Biol. Macromol. 127, 544-554.

[8]Gao, C., Zhang, X., Wang, Y., 2019. Preparation and properties of a novel lignin-based polyurethane slow-release fertilizer. J. Appl. Polym. Sci. 136, 47812.
